# Direct Exfoliation of Natural SiO_2_-Containing Molybdenite in Isopropanol: A Cost Efficient Solution for Large-Scale Production of MoS_2_ Nanosheetes

**DOI:** 10.3390/nano8100843

**Published:** 2018-10-17

**Authors:** Wenyan Zhao, Tao Jiang, Yujie Shan, Hongrui Ding, Junxian Shi, Haibin Chu, Anhuai Lu

**Affiliations:** 1 School of Chemistry and Chemical Engineering, Inner Mongolia University, Hohhot 010021, Inner Mongolia, China; zhaowenyan@imu.edu.cn (W.Z.); Jiang.T@mail.imu.edu.cn (T.J.); shanyj930@163.com (Y.S.); chuhb@imu.edu.cn (H.C.); 2 School of Earth and Space Sciences, Peking University, Beijing 100871, China; DHR@pku.edu.cn; 3 School of Ecology and Environment, Inner Mongolia University, Hohhot 010021, Inner Mongolia, China

**Keywords:** natural molybdenite, MoS_2_ nanosheet, SiO_2_, liquid exfoliation, photoelectric properties

## Abstract

The cost-effective exfoliation of layered materials such as transition metal dichalcogenides into mono- or few- layers is of significant interest for various applications. This paper reports the preparation of few-layered MoS_2_ from natural SiO_2_-containing molybdenite by exfoliation in isopropanol (IPA) under mild ultrasonic conditions. One- to six-layer MoS_2_ nanosheets with dimensions in the range of 50-200 nm are obtained. By contrast, MoS_2_ quantum dots along with nanosheets are produced using N-methyl-pyrrolidone (NMP) and an aqueous solution of poly (ethylene glycol)-block-poly (propylene glycol)-block-poly (ethylene glycol) (P123) as exfoliation solutions. Compared with molybdenite, commercial bulk MoS_2_ cannot be exfoliated to nanosheets under the same experimental conditions. In the exfoliation process of the mineral, SiO_2_ associated in molybdenite plays the role of similar superfine ball milling, which significantly enhances the exfoliation efficiency. This work demonstrates that isopropanol can be used to exfoliate natural molybdenite under mild conditions to produce nanosheets, which facilitates the preparation of highly concentrated MoS_2_ dispersions or MoS_2_ in powder form due to the volatility of the solvent. Such exfoliated MoS_2_ nanosheets exhibit excellent photoconductivity under visible light. Hence, the direct mild exfoliation method of unrefined natural molybdenite provides a solution for low-cost and convenient production of few-layered MoS_2_ which is appealing for industrial applications.

## 1. Introduction

Since graphene’s initial discovery [1], two-dimensional (2D) transition metal dichalcogenide semiconductors (TMD) have attracted great research interest for their unique electronic and optical characteristics, which are distinctively different from those of their bulk materials [2,3,4,5,6]. MoS_2_ is the most popular member of the series, which compensates for the disadvantage caused by the absence of a band gap of graphene, and shows considerable anisotropy due to its large intrinsic band gap, resulting in novel electronic, optical, mechanical, and structural characteristics [7,8,9,10,11]. In many applications, such as batteries, composites, sensors, and catalytic activities, MoS_2_ needs to be produced on a large-scale, and preferably at a lower cost.

Different methods to prepare monolayer and few-layered MoS_2_ have been developed, including mechanical exfoliation [12], liquid-phase exfoliation [13,14,15], chemical exfoliation [16,17], and chemical vapor deposition [18,19]. Generally, studies have found that liquid-phase exfoliation has great potential for scalable production of 2D materials. Coleman et al., have indicated that layered materials can be exfoliated into ultrathin-layered 2D nanomaterials in organic solvents by sonication [14]. But for large-scale applications, it has been difficult to achieve high enough concentrations. In many cases, the exfoliating media used with high boiling points are difficult to remove. A method of mixed-solvent containing volatile solvents has been used to exfoliate TMD [20,21,22]. For example, the mixture of water and ethanol was demonstrated to be an effective solvent for the exfoliation of MoS_2_ nanosheets [23]. Nguyen et al. have reported a two-solvent grinding-assisted liquid phase exfoliation of layered MoS_2_, avoiding the solvent residue [24]. Ultrasonic treatment is widely used in liquid exfoliation. Ultrasonic force generates acoustic cavitation. The shear forces coming from acoustic cavitation can break the Van der Waals interactions of bulk materials, leading to the exfoliation of 2D materials [14,24], and affecting the structural characteristics of nanoparticles [25]. In many cases, ultrasound probes are utilized to exfoliate 2D materials. Their use can generate violent mechanical driving forces by concentrating energy in a small amount of dispersion liquid. In contrast, an ultrasonic water bath is milder and more cost-effective alternative, which is appealing for samples batch processing. However, it is rarely used in current liquid exfoliation methods.

The intercalation of various intercalates is another useful liquid phase exfoliation method to weaken neighboring layers arising from the interlayer expansion. Peng et al. recently demonstrated a lithium-intercalated single-crystals exfoliation method for 2D TMD nanomaterials in water by only simple manual shaking [26]. However, this chemical exfoliation method partly leads to structural deformation, and is very sensitive to the environmental conditions. To overcome the aforementioned drawback, an alternative approach used grinding/sonication-assisted Li^+^ intercalation in simulated sun irradiation conditions, avoiding the use of hazardous liquids such as butyllithium; however, the use of N-methyl-pyrrolidone (NMP) leads to persistent residues on the exfoliated flakes [27]. Great effort has been made to develop new exfoliation technology. Recently, a microcentrifugation surface acoustic wave device was developed to exfoliate MoS_2_, which applied an electric field and mechanical shear force for fast and efficient exfoliation. Similarly, the surfactant residues and expensive equipment defeat the purpose [28]. Consequently, it remains challenging to develop liquid exfoliation techniques in more convenient and cost-effective ways to obtain highly-concentrated 2D MoS_2_ dispersions that allow separation as precipitates for further application.

In many cases, the preparation of 2D MoS_2_ in the lab is based on the liquid exfoliation of MoS_2_ powder and single crystals. Savjani et al., [29] and Dong et al., [30] have produced MoS_2_ nanosheets by directly exfoliating molybdenite minerals in NMP, and showed that unrefined molybdenite could be an exfoliation source of 2D materials. However, in many cases, the molybdenite powders are purified before exfoliation, and the effects of the impurity constituents on the exfoliation have not been considered. Because natural molybdenite contains quartz, it would thus be of interest to learn whether the quartz in natural molybdenite may play the role of ball mill during the exfoliation of molybdenite, before being separated from the suspension of the MoS_2_ nanosheets after exfoliation. Furthermore, we wonder if a solvent whose surface tension does not harshly match with the layered bulk materials can effectively exfoliate natural molybdenite under mild conditions. Relevant studies should be performed to solve these questions.

Here, we prepared 2D MoS_2_ nanomaterials by liquid exfoliation of SiO_2_-containing molybdenite ores in the volatile solvent, isopropanol (IPA), as well as in NMP, a surfactant aqueous solution under mild water bath ultrasound conditions. The nanomaterials obtained from molybdenite have been compared with the products obtained from commercial MoS_2_ powder in the same experimental conditions in terms of exfoliation efficiency, composition, structural features, and photoelectric properties.

## 2. Materials and Methods 

### 2.1. Materials

Natural molybdenite (NM) was collected and enriched in Dasuji diggings, Zhuozi County, Inner Mongolia. Compositional analysis by X-ray fluorescence (XRF) spectrometry shows that natural molybdenite mainly consisted of Mo, S, SiO_2_, Al_2_O_3_ (Table S1). The exact content of Mo is 49.71wt%, S is 27.65wt%, and SiO_2_ is 14.15wt%, which was determined using an inductively-coupled plasma-mass (ICP-MS) spectrometer. The NM was ball-milled (XGB planetary mill, 100 stainless steel balls of 6 mm diameter, rotation speed 500 rpm) for 1 h and sifted to obtain mineral powder with particle sizes of <45 μm. The scanning electron microscope (SEM) images are shown in Figure S1a,b. The X-ray diffraction (XRD) patterns prove that the NM was mainly formed from 2H MoS_2_ and quartz-phase SiO_2_ (Figure S2). Energy dispersive spectrometry (EDS) shows that the SiO_2_ and MoS_2_ are mixed uniformly on the micro-scale (Figure S3). Commercially-available MoS_2_ (CM, 99%, average 2 μm) was bought from Sigma-Aldrich (Figure S1c,d). NMP, P123, and IPA were purchased from J&K Chemicals (Beijing, China).

Preparation of control samples of NM: SiO_2_ associated in NM was removed by HF method; 2 g of NM was added to 20 mL mixed acid solution (16 mL 40%HF and 4 mL H_2_SO_4_) in 50 mL Teflon crucible, and maintained under magnetic stirring for 3 h at 80 °C; after reaction, the product was filtrated, washed with Milli-Q water several times to neutral, then dried at 60 °C in oven.

### 2.2. Exfoliation Process

0.5 g of NM or CM was added to 50 mL of exfoliation solution (IPA, NMP or P123 aqueous solutions) in a 100 mL glass vial. The mixture was batch sonicated for 16 h in an ultrasonic water bath with a frequency of 40 kHz (volume 15 L, power 400 W), taking care to have the water level higher than the level of the suspension. The ultrasonic temperature was controlled below 50 °C. The resulting dispersion was centrifuged for 15 min at 10,000 rpm to remove the very thick nanosheets and excess impurity. The supernatants were collected by pipette. The products derived from NM or CM were labeled as NM-X or CM-X respectively, where X is the exfoliation solvent.

### 2.3. Characterization

Compositional analysis of NM was achieved using a Rigaku ZSX Primus II XRF spectrometer and a Thermo X Series II ICP-MS spectrometer. The UV-vis (Ultraviolet-visible) spectra were performed by a Hitachi UV-3900 spectrometer using 1.0 cm quartz cuvette. Accurate dilutions of the dispersions were produced to obtain suitable UV-vis spectra. The XRD pattern measurements were carried out using a PANalytical X’Pert Pro diffractometer with Cu Kα radiation at 0.15406 nm (equipped with monochromator). Raman spectra were collected by a MicroRaman spectrometer (Renishaw in Via Reflex, Gloucestershire, UK), equipped with a 532 nm DPSS laser and a 2400  lines/mm grating. For the specimen preparation, the suspension was drop-casted onto a silicon substrate (wafer) and heated to 50 °C to form an opaque film. SEM imaging was performed using Hitachi S4800. Energy dispersive X-ray analyses were performed using Bruker QUANTAX 200 energy dispersive spectrometer. The transmission electron microscopy (TEM) images were measured using a FEI Tecnai G2F20S-TWIN. Samples for TEM imaging were prepared by drop-casting the 2D MoS_2_ dispersion onto 200 mesh lacey carbon-coated copper grid and dried before analysis. Nanosheets thickness measurements were carried out on a Bruker  atomic force microscopy (AFM) (Dimension Icon, Bruker, Billerica, MA, USA) operating in ScanAsyst mode. A silicon nitride probe (type SCANASYST-AIR, Bruker, Billerica, MA, USA) with a curvature radius of 2 nm was used for AFM measurements. Its specifications include a force constant of approximately 0.4 N/m, and a resonant frequency of 70 kHz. Samples for AFM were prepared by diluting the samples in ethanol to a concentration in the range of 100 μg/mL, drop-casting the diluted suspension onto a clean silicon wafer piece, and heating to evaporate the solvent.

### 2.4. Photoelectrochemical Measurement

The working electrodes were prepared on glassy carbon (GC) electrodes, which were dip-coated with nano MoS_2_ dispersions (loading 18 μg/cm^2^). The electrochemical measurements were tested in 0.5 M Na_2_SO_4_ with a conventional three-electrode system. Pt plate and saturated Ag/AgCl were used as counter electrode and reference electrode, respectively. The photocurrents were measured on a CHI760E Chenhua electrochemical workstation. The visible light irradiation source was obtained by a 300 W Xe lamp (Beijing Trustech, Beijing, China, PLS-SXE300c) with a 420 nm cut-off filter. 

## 3. Results and Discussion

**Figure 1:**
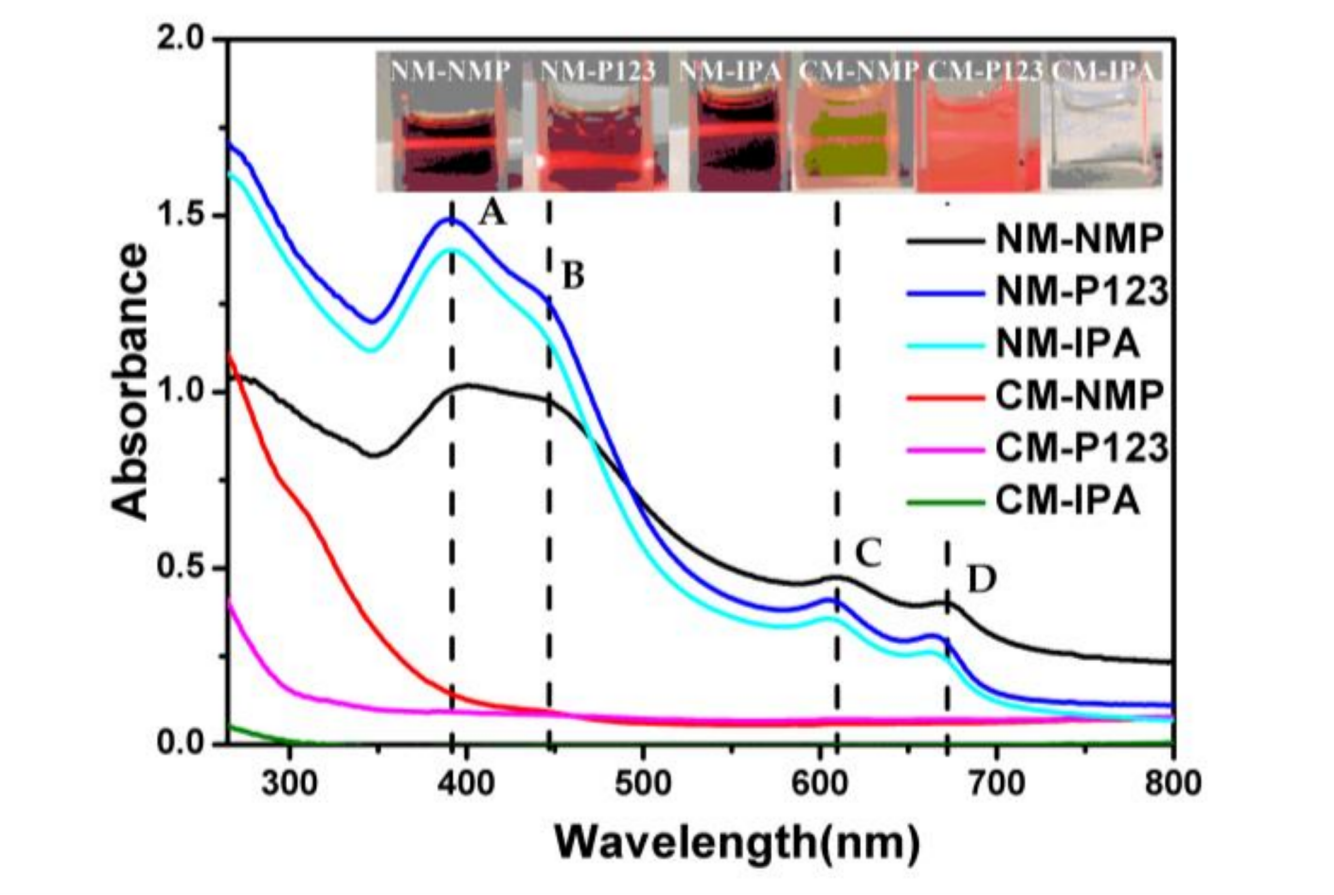
Ultraviolet-visible absorption spectra of products exfoliated in various solvents from natural molybdenite (NM) and commercial MoS_2__(_CM). The Photographs of different products with a red laser crossed through is shown in the inset.

Nano MoS_2_ dispersions were prepared from NM or CM using a simple bath sonication technique in IPA, NMP, or aqueous solutions of surfactant P123. After bath sonication for 16 h, suspension of different colours were obtained and labeled as NM-NMP, NM-P123, NM-IPA, CM-NMP, CM-P123, and CM-IPA respectively (Figure 1 inset). Products from NM are dark green, CM-NMP is golden yellow, CM-P123 is yellow-green, and CM-IPA is colourless and transparent. Except for CM-IPA, the Tyndall effect was observed, which indicates that the suspension was a colloidal dispersion. UV-vis spectra of the exfoliated MoS_2_ are shown in Figure 1. In the samples derived from NM, the characteristic absorption bands of MoS_2_ nanosheets at 390nm (A), 450nm (B), 610nm (C), and 670 nm (D) are clearly observed [31,32,33]. The excitonic peaks at 610 nm and 670 nm arise from the K point of the Brillouin zone. The peaks at ~390 nm and ~450 nm can be attributed to the direct transition from the deep valence band to the conduction band [34,35]. But in the samples derived from CM, absorption bands in the visible light region are very weak. These results from UV-vis absorption only confirm that NM can be exfoliated to produce MoS_2_ nanosheets in three solvents, but CM cannot be exfoliated to obtain nanosheets under mild ultrasonic conditions. 

**Figure 2:**
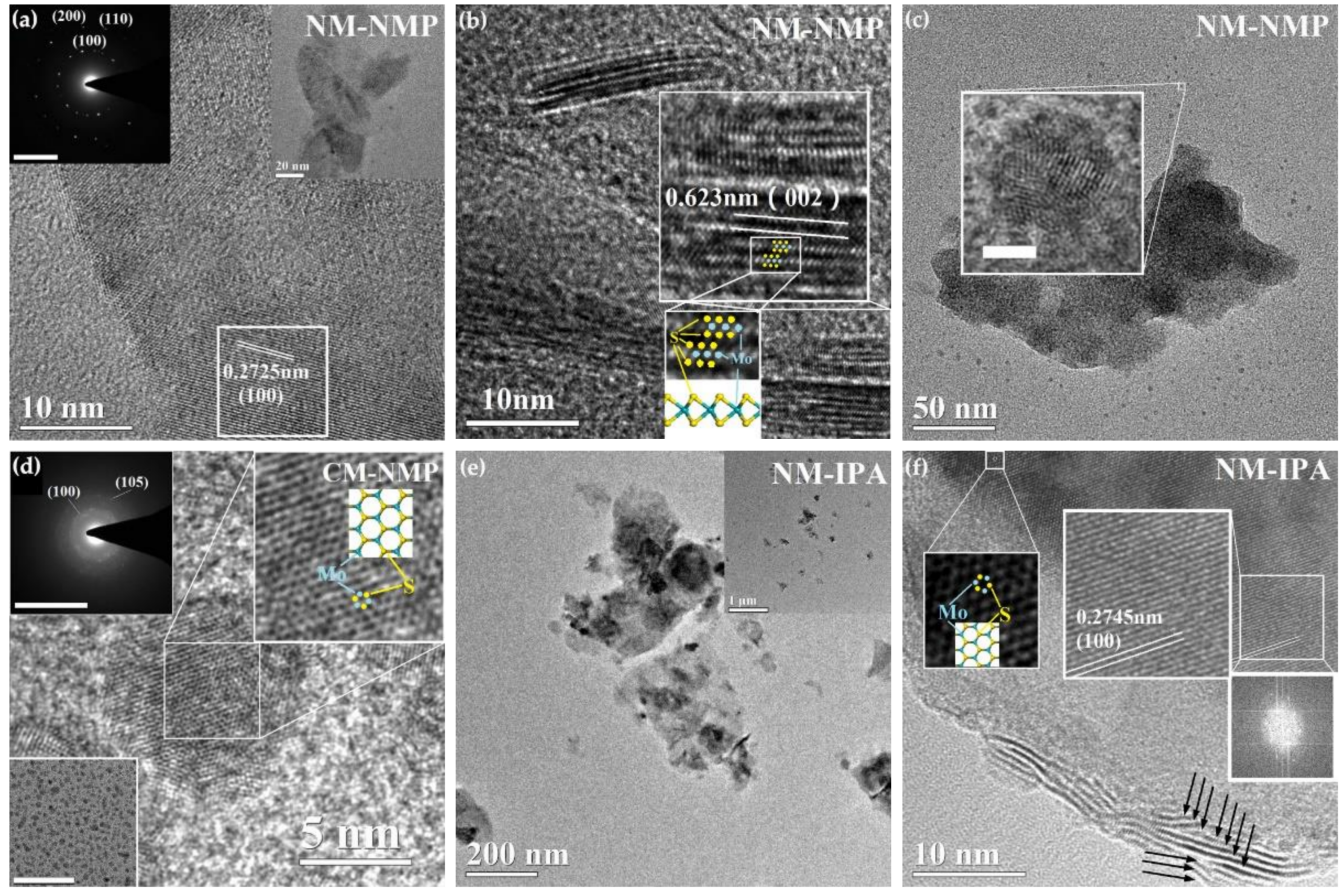
The high-resolution transmission electron microscopy (HRTEM) images of products exfoliated in N-methyl-pyrrolidone (NMP) from NM (**a**, **b**, **c**), CM (**d**), and products exfoliated in isopropanol (IPA) from NM (**e**, **f**). Insets in (**a**): a selected area electron diffraction (SAED) pattern, scale bar = 5 nm^-1^, a low resolution image, scale bar = 20 nm; insets in (**b**, **f**): magnification of the selected area and schematic diagram of atomic arrangement; inset in (**c**): a quantum dot image, scale bar =2 nm; insets in (**d**): a SAED pattern, scale bar = 10 nm^-1^, a low resolution image, scale bar = 50 nm, magnification of the selected area and schematic diagram of atomic arrangement; inset in (**e**): a low resolution image, scale bar = 1 µm.

**Figure 3:**
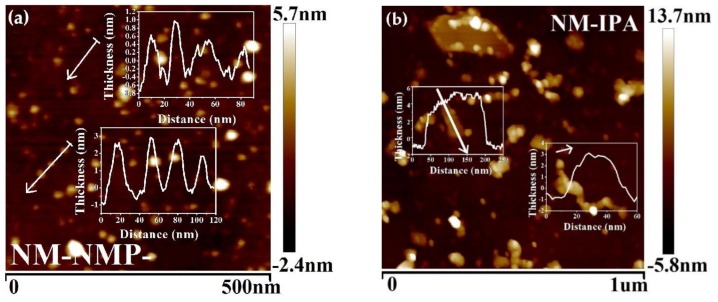
Atomic force microscopy (AFM) images of products exfoliated from NM in NMP (**a**) and in IPA (**b**). The insets show the height profiles along with the arrows. AFM images and height profile confirming the thickness of <6 nm.

Further characterization was studied using the TEM images of exfoliated MoS_2_ for NM-NMP, CM-NMP, and NM-IPA. TEM images of NM-NMP are shown in Figure 2a,b,c. The transparency of the nanosheets confirms that the MoS_2_ sheets are very thin. Clear lattice fringes with a separation of 0.2725 nm are observed for (100) planes of 2H-MoS_2_ in Figure 2a. The selected area electron diffraction (SAED) pattern of MoS_2_ nanosheets shown in the left inset of Figure 2a reveals the highly crystalline nature of the sheets with a hexagonal diffraction pattern. The diffraction spots shown by the SAED correspond to the (100), (110), (200) crystal faces of the 2H-MoS_2_. Figure 2b shows several erected nanosheets. The nanosheets have only a few molecular layers, with interlayer spacing of 0.623 nm corresponding to the (002) crystal plane; the atomic arrangements are shown in Figure 2b inset. In addition, MoS_2_ quantum dots around the nanosheets are clearly observed in Figure 2c. Figure 2d shows the image of CM-NMP, in which quantum dots of ~5nm are observed. The left inset of Figure 2d shows SAED pattern of quantum dots which correspond to the (100), (105) crystal faces of hexagonal system, and the inset at the top right corner shows the hexagonal arrangement of 2H-MoS_2_. The above results indicate that MoS_2_ quantum dots, along with nanosheets, were obtained by exfoliating NM; meanwhile, only quantum dots were produced from CM powder in NMP. TEM images of NM-IPA are shown in Figure 2e,f. MoS_2_ nanosheets with lateral size ~100 nm are observed (Figure 2e), and the layer structure of the edge warpage can be clearly shown in Figure 2f (arrow indication). Lattice fringes with a separation of 0.2745 nm are responsible for (100) planes of 2H-MoS_2_, and the inset at the top left corner shows the hexagonal arrangement of 2H-MoS_2_. MoS_2_ nanosheets are the only products observed in NM-IPA. The majority of the exfoliated MoS_2_ nanosheets are close to 1–6 layers thickness. More images for the other products obtained, such as NM-P123 and CM-P123, can be found in Figure S4. Quantum dots spreading nanosheets of MoS_2_ were observed in NM-P123 (Figure S4a,b), and amounts of quantum dots and a few nanosheets were observed in CM-P123 (Figure S4c,d). To further ascertain the morphology and thickness of exfoliated MoS_2_, AFM was carried out. Figure 3 shows scan AFM images in ScanAsyst mode of MoS_2_ nanosheets in NM-NMP and NM-IPA samples. Figure 3a,b reveals that the thickness of nanosheets was less than 6 nm both in NM-NMP and NM- IPA. Given that the thickness of a MoS_2_ monolay is ~1 nm [14,23,36], this suggests that the obtained MoS_2_ nanosheets contain ~1 to 6 layers, which agrees with the TEM results.

**Figure 4:**
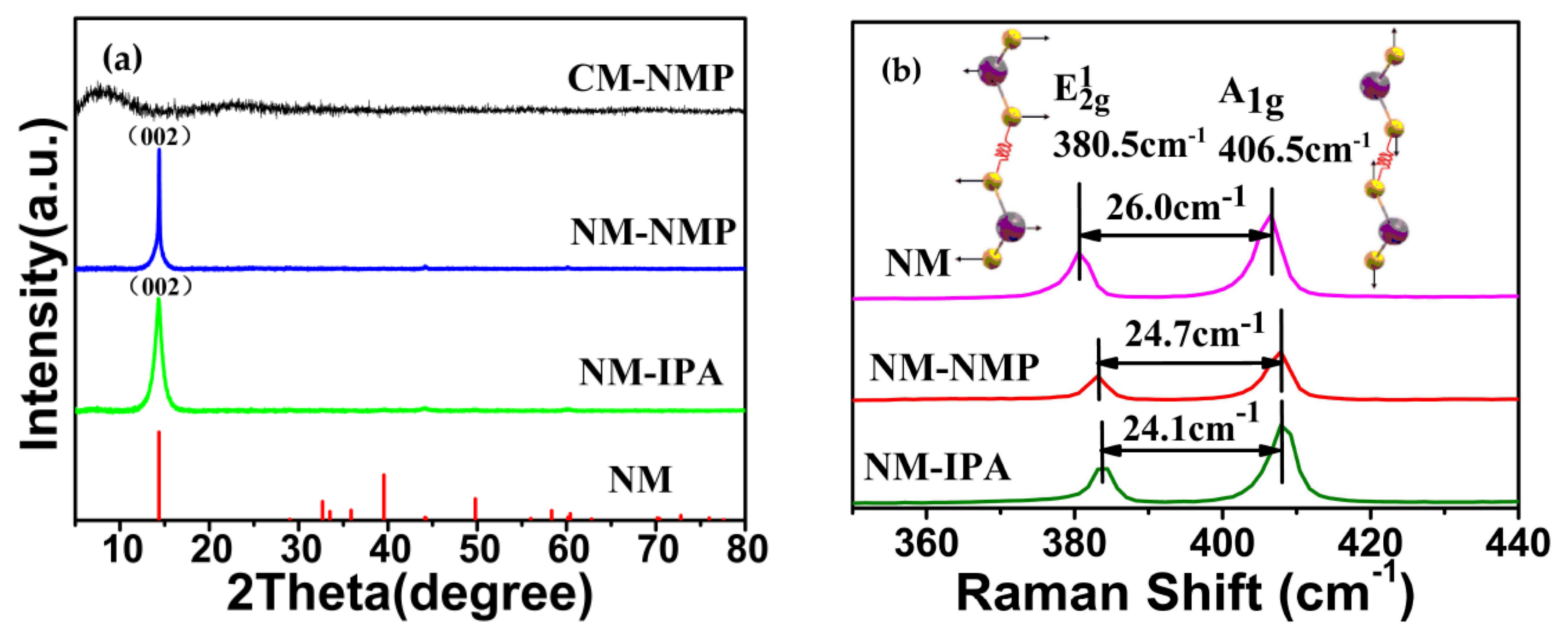
(**a**) X-ray diffraction (XRD) patterns of NM-NMP, NM-IPA, CM-NMP and bulk NM. (**b**) Raman spectra of bulk NM, NM-NMP and NM-IPA.

To further confirm the phase, XRD and Raman spectroscopy were performed (Figure 4). Due to the presence of polymeric surfactant P123, we could not use AFM, XRD, or Raman to analyze product NM-P123 unless the samples were treated by washing or calcining, which could change the samples (Figure S5 and S6). The appearance of (002) reflection in the XRD patterns of NM-NMP and NM-IPA indicates the presence of MoS_2_ nanosheets derived from NM with good crystallinity (Figure 4a). Meanwhile, XRD pattern of CM-NMP exhibiting no apparent reflection also indicates that CM-NMP mainly consists of quantum dots. Raman spectra are shown in Figure 4b. The characteristic shifts near 380.5 cm^-1^ and 406.5 cm^-1^ for bulk NM, NM-NMP, and NM-IPA respectively correspond to E^1^_2g_ and A_1g_ vibrations modes of Mo-S bands in 2H MoS_2_ [37]. After exfoliation, the E^1^_2g_ and A_1g_ peak shift, and the shift difference between E^1^_2g_ and A_1g_, is generally only changed with the layer number of molecules. So, the reduction of the shift difference between the two peaks can be considered to be an indicator of the layer number of MoS_2_ nanosheets [38]. Compared to 26.0 cm^-1^ shift difference of the two characteristic peaks for bulk NM, the shift difference reduces to 24.7 cm^-1^ for NM-NMP and 24.1 cm^-1^ for NM-IPA respectively. On the basis of information provided by Li et al., [37], this result indicates that the thickness of MoS_2_ nanosheets exfoliated from NM was mostly less than 6 layers, which is consistent with the measurements of TEM and AFM.

MoS_2_ concentration in suspension was measured by atomic absorption spectroscopy. The concentrations of dispersed nano MoS_2_ exfoliated from NM are 596, 485, and 252 mg/L for P123, NMP, and IPA solution respectively. Compared to the concentrations of products exfoliated from CM, which are listed in Table 1, the concentration of MoS_2_ derived from NM is relatively high. Furthermore, nanosheets are more easily obtained from NM. The surprise result raises a question: why can NM with lower purity and larger particle sizes compared with CM be exfoliated to produce more nanosheets? In many exfoliation methods, MoS_2_ nanosheets were obtained using an ultrasound probe as a violent mechanical driving force because it concentrates more energy [14,36,39]. In our case, due to the dispersive energy of the ultrasonic water bath, MoS_2_ quantum dots separated from the crystal defects were the only product from CM [40]. However, MoS_2_ nanosheets were obtained under the same mild ultrasound conditions from NM. NM contains small amounts of impurities compared with CM, mainly SiO_2_ (Table S1). Thus, it seems reasonable to hypothesize that the quartz phase SiO_2_ in the NM may cause collisions with MoS_2_ during bath sonication, leading to cleavage of bulk layers into ultrathin sheets, which can play the role of superfine ball milling. Furthermore, compared with the added ball milling medium (e.g., ZrO_2_) [39], associated quartz in the NM bonds with MoS_2_ more closely and uniformly, and the particle size of that is much smaller, thereby causing more effective stripping. After exfoliation, most of the SiO_2_ was removed from dispersion by centrifugation, as observed in the EDS. Quantitative analysis shows that Si content in exfoliated nanosheets is 0.32wt%, which is much lower than that in natural molybdenite (5.27wt%). EDS analysis shows Mo : S to be 1 : 2 in all case, and reveals that the nano MoS_2_ prepared from NM does not contain more non-negligible impurity contaminations than that obtained from CM.

**Scheme 1:**
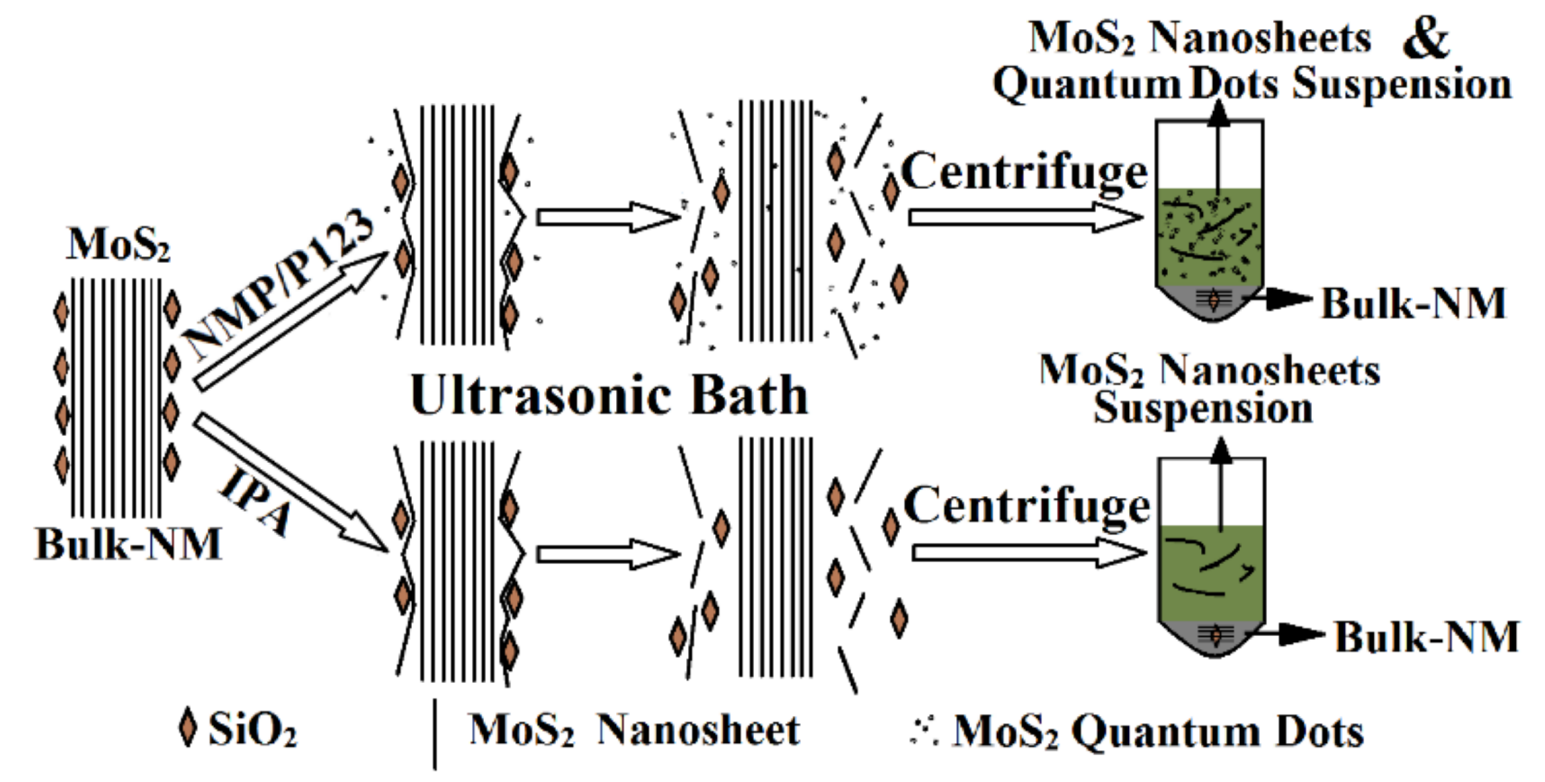
Exfoliation mechanism of natural SiO_2_-associated molybdenite in IPA, NMP, and P123 aqueous solution, under mild ultrasonic condition.

To test the aforementioned hypotheses, we prepared control samples of NM which were purified to remove SiO_2_ by the HF method, and compared them with the initial NM as the source powders of exfoliation in IPA. After HF treatment, the SiO_2_ content in molybdenite reduces from 11% to 0.65%, and the absorbance intensity of exfoliation products at ca. 670 nm plummets, as shown in Figure S7, indicating that the yield of the nanosheets from NM with SiO_2_ removed is considerably lower than that from initial NM. Therefore, this result supports our argument about the associated SiO_2_ dependent exfoliation mechanism of the mineral, as shown in Scheme 1.

In addition, the exfoliation efficiency varies greatly with the solvents, resulting in a different morphology and yield of exfoliated nano-MoS_2_. During the wet grinding process, the choice of grinding solvent has a significant influence on the final product [24]. According to the principle of matching surface energy of solvents [41], NMP and 10 wt% P123 with a surface tension close to 40 mN/m are good solvents for exfoliating bulk MoS_2_; therefore, MoS_2_ quantum dots are easy to obtain by water bath sonication in NMP or P123. As shown in the height profile of AFM (Figure 3), NMP produces thinner nanosheets than IPA. Studies have suggested that a close match of the surface energy between the solvent and the layered material leads to the exfoliation of flakes with increased aspect ratios [42]. Thinner flakes are more liable to breakdown and produce quantum dots by ultrasonic vibration resulting from the ultrasonic water bath. Because IPA surface tension is 20.80 mN/m, far away from 40 mN/m, nano MoS_2_ is not successfully prepared in IPA from CM by mild water bath sonication; however, it can be prepared from NM. Due to the surface tension of IPA not being matched, the quantum dots cannot be exfoliated off from the crystal defects, but MoS_2_ nanosheets have been achieved in IPA via a mineral-associated quartz milling process. Though the concentrations of dispersed MoS_2_ nanosheets in IPA are relatively low, the highly concentrated MoS_2_ nanosheets dispersion or its powder form can be obtained due to the volatility of IPA, and the solvent can be recovered for recycling exfoliation to improve yields. Therefore, aggregation of MoS_2_ nanosheets caused by extracting exfoliated MoS_2_ from NMP via heating evaporation at high temperature can be avoided. Thereafter, the obtained powder from IPA is convenient for characterization and preparation of samples in applications avoiding the effect of a solvent, which could be widely applied.

**Figure 5:**
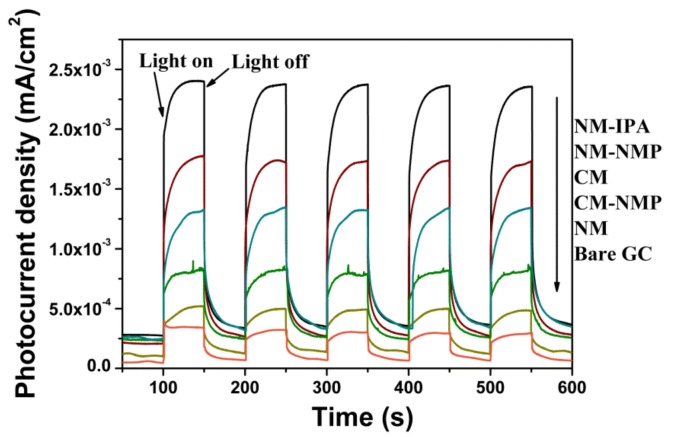
The transient on/off photocurrent responses vs. time plotted for NM-IPA, NM-NMP, CM-NMP, NM, CM, and bare GC in 0.01 M Na_2_SO_4_ solution under visible-light.

Two-dimensional MoS_2_ has shown prior promise for various energy-related applications because of its intrinsic band-gap structure. To confirm the photoelectric properties of our exfoliated MoS_2_, simple light-to-electric conversion experiments were conducted. The time-dependent photocurrent of exfoliated nano MoS_2_ was measured at 0.6 V bias voltage under alternating darkness and visible light conditions. As shown in Figure 5, all the samples exhibited repeatable and stable responses to illumination. In contrast, the photocurrents of NM-IPA and NM-NMP are higher than those of bulk NM and CM, and the highest ΔI is generated by NM-IPA, which is around 9-fold higher than that of bare GC. The remarkable increase in photocurrent density results from the sensitivity of exfoliated MoS_2_ nanosheets to visible light and the efficient separation of photo-generated electron-hole pair. Unexpectedly, a lower photocurrent response of CM-NMP was observed, which is close to that of bare GC. A higher photocurrent response of MoS_2_ nanosheets (NM-IPA) in comparison to their bulk structure gives rise to the change in band structure from indirect to direct bandgap [43,44]. The remarkable decrease in the photocurrent density of CM-NMP (MoS_2_ quantum dots) was observed because of its insensitivity to visible light and the quantum confinement effect.

## 4. Conclusions

Few-layered MoS_2_ was prepared from natural SiO_2_-containing molybdenite by exfoliation in IPA under mild ultrasonic conditions. The exfoliation products from NM and CM in different exfoliation solvents including IPA, NMP, and aqueous solutions of P123 were compared. The results show that the yield of nano MoS_2_ exfoliated from the molybdenite is higher than that from CM powder. The choice of exfoliation solvent plays a crucial role. IPA can successfully exfoliate molybdenite to produce nanosheets, but cannot exfoliate commercial MoS_2_ powder. NMP and aqueous solutions of P123 can exfoliate NM to MoS_2_ nanosheets and quantum dots, and exfoliate CM to quantum dots. It was found that the MoS_2_ nanosheets produced from NM in IPA exhibit greater photocurrent density under visible light. The quartz phase associated in molybdenite is a major contributor to the effective exfoliation, and plays the role of superfine ball milling. 

This work highlights that unrefined natural SiO_2_-containing NM can be used as an exfoliation source of 2D MoS_2_; the ability to use volatile IPA to exfoliate it will enable recirculated exfoliation to produce MoS_2_ nanosheets with higher yields, for cost–effective and large-scale industrial applications. However, in the exfoliation process of minerals, the solvent has an influence on the morphology of the product, which is poorly understood at present. Further research may lead to proper evaluations of the effects of different solvents on exfoliation, which help us to fully elucidate the underlying exfoliation mechanisms.

## Figures and Tables

**Table 1:** The concentrations and morphologies of MoS_2_ exfoliated.

	NM-NMP	NM-P123	NM-IPA	CM-NMP	CM-P123	CM-IPA
Concentration of MoS_2_ (mg/L)	485	596	252	239	485	―^1^
Morphology of MoS_2_	Nanosheets & quantum dots	Nanosheets & quantum dots	Nanosheets	quantum dots	Nanosheets & quantum dots	―^1^

^1^ No detection.

## Supplementary Materials

The following are available online at http://www.mdpi.com/2079-4991/8/10/843/s1, Figure S1: SEM images of natural molybdenite (a), sifted natural molybdenite after ball-milled (b) and commercial MoS_2_ (c, d); Figure S2: XRD patterns of natural molybdenite and commercial MoS_2_; Figure S3: Elemental analysis of natural molybdenite using EDS shows the SiO_2_ and MoS_2_ are mixed uniformly in the micro-scale; Figure S4: TEM images of products exfoliated in P123 aqueous solution from natural molybdenite (a, b), commercial MoS_2_ (c, d); Figure S5: (a) XRD patterns of products exfoliated in P123 from natural molybdenite and commercial MoS_2_, (b) TEM images of NM-P123 powder-sample obtained by washing with deioned water several times to remove P123 after centrifuging at 1500rpm for 45min; Figure S6: (a) Raman spectrum of NM-P123 powder-sample obtained by calcining at 450°C for 2h, (b) TGA curves of NM, CM, NM-IPA-Powder and P123; Figure S7: UV-Vis absorption spectra stack plot of MoS_2_ dispersions obtained from the initial NM and NM removed SiO_2_, Table S1: Content of natural molybdenite by component analysis using XRF.
