# Supplementary material for: Direct Exfoliation of Natural SiO2-Containing Molybdenite in Isopropanol: A Cost Efficient Solution for Large-Scale Production of MoS2 Nanosheetes
Source: Nanomaterials (Basel). 2018 Oct 17;8(10):843. doi: 10.3390/nano8100843 (PMC6215130; doi:10.3390/nano8100843)
Supplement: Supplementary file 1 [file nanomaterials-08-00843-s001.pdf]

Supplementary materials

Direct Exfoliation of Natural SiO_2-Containing Molybdenite in Isopropanol: A Cost Efficient Solution for Large-Scale Production of MoS_2 Nanosheets

Wenyan Zhao [1], Tao Jiang [1], Yujie Shan [1], Hongrui Ding [3], Junxian Shi [2,*], Haibin Chu [1], Anhuai Lu [3,*]

[1] School of Chemistry and Chemical Engineering, Inner Mongolia University, Hohhot, 010021, Inner Mongolia, PR China

[2] School of Ecology and Environment, Inner Mongolia University, Hohhot, 010021, Inner Mongolia, PR China.

[3] School of Earth and Space Sciences, Peking University, Beijing, 100871, PR China

*Corresponding author: E-mail: 111969116@imu.edu.cn (J.X. Shi), ahlu@pku.edu.cn (A.H. Lu).

Table S1: Content of natural molybdenite by component analysis using XRF

Element	Mo	K_2O	CaO	Mn	Sr	Na_2O	Sn	Ge	Yb	Y	As
Content/10^{-2}（wt%）	58	1	0.4	0.05	0.008	<0.01	<0.004	<0.002	<0.002	<0.001	<0.0008
Element	S	Fe_2O_3	Zn	Ni	F	Hg	Ti	In	Hf	Te	Co
Content/10^{-2}（wt%）	21	1	0.2	0.02	<0.05	<0.01	<0.003	<0.002	<0.002	<0.001	<0.0007
Element	SiO_2	Pb	MgO	Bi	Cd	Sb	Th	La	Ta	W	Zr
Content/10^{-2}（wt%）	11	1	0.1	0.01	<0.02	<0.009	<0.003	<0.002	<0.002	<0.001	<0.0006
Element	Al_2O_3	C	Cr	Rb	Ba	Ag	V	Ce	Cu	Ga	Nb
Content/10^{-2}（wt%）	2	1	0.06	0.009	<0.02	<0.006	<0.002	<0.002	<0.001	<0.0008	<0.0005

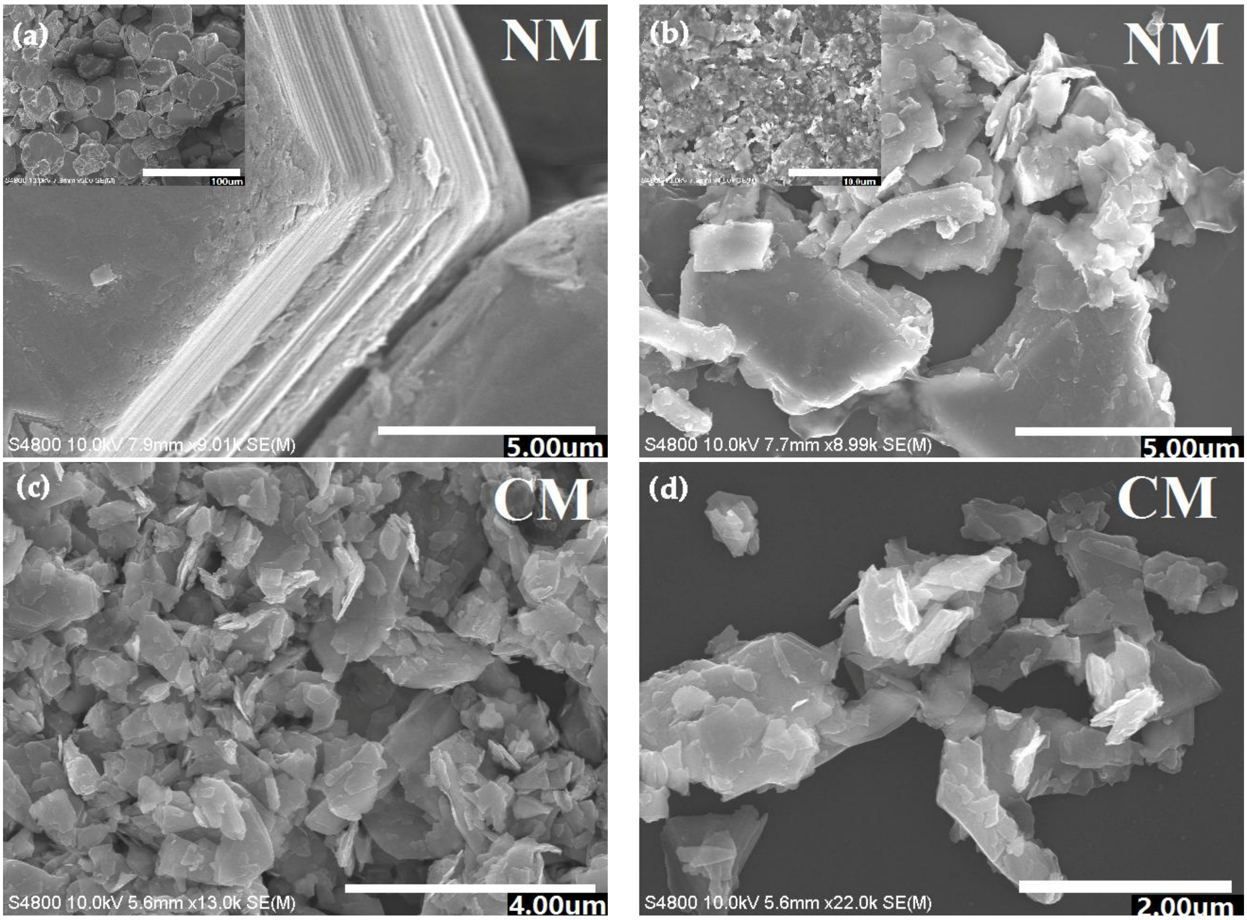

Figure. S1: SEM images of natural molybdenite (a), sifted natural molybdenite after ball-milled (b) and commercial MoS_2 (c, d).

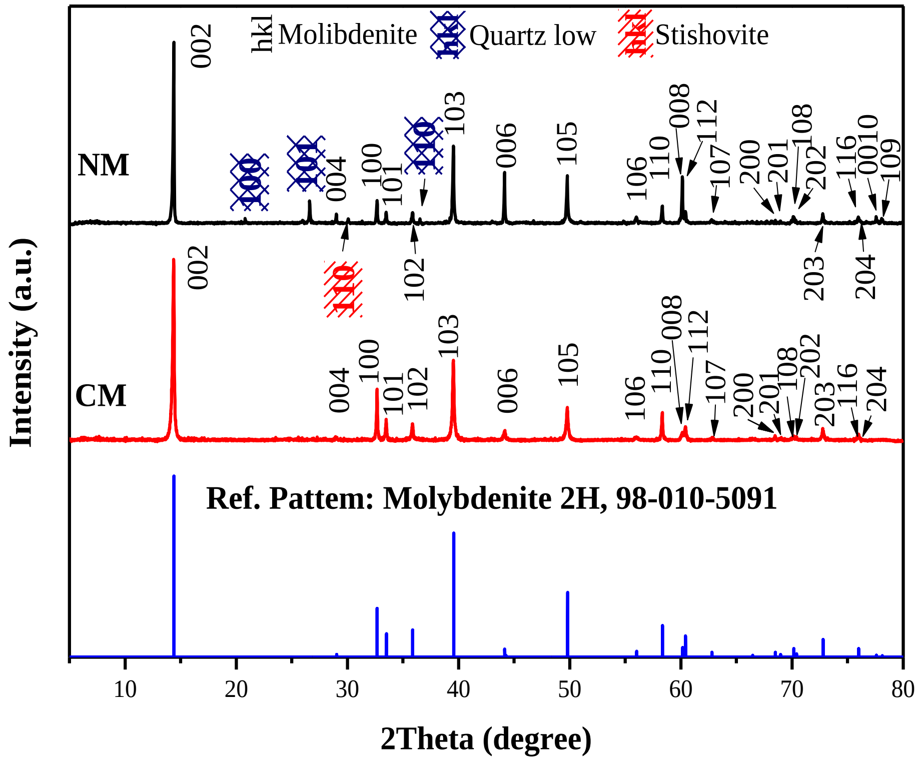

Figure. S2: XRD patterns of natural molybdenite and commercial MoS_2 shows both raw material mainly consist of 2H MoS_2 and the presence of quartz phase SiO_2 in natural molybdenite.

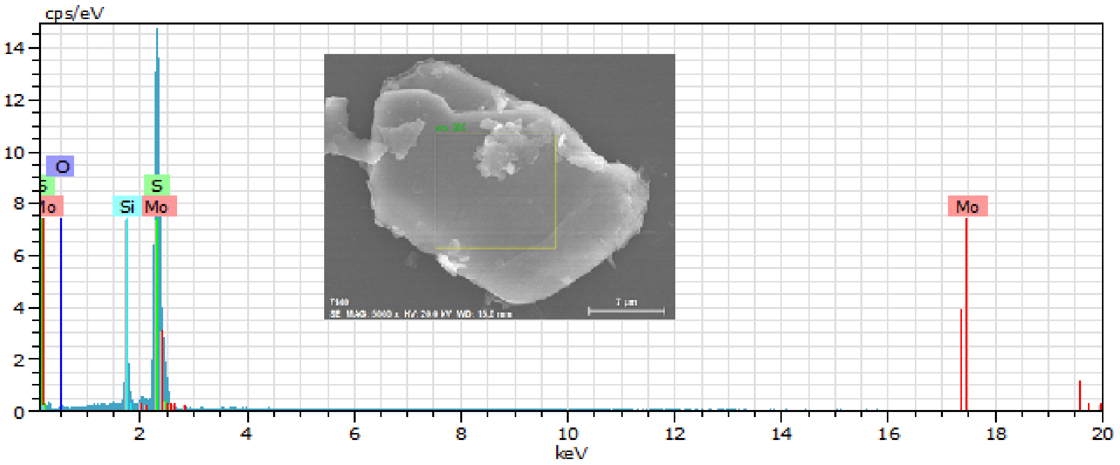

Fig. S3: Elemental analysis of natural molybdenite using EDS shows the SiO_2 and MoS_2 are mixed uniformly in the micro-scale.

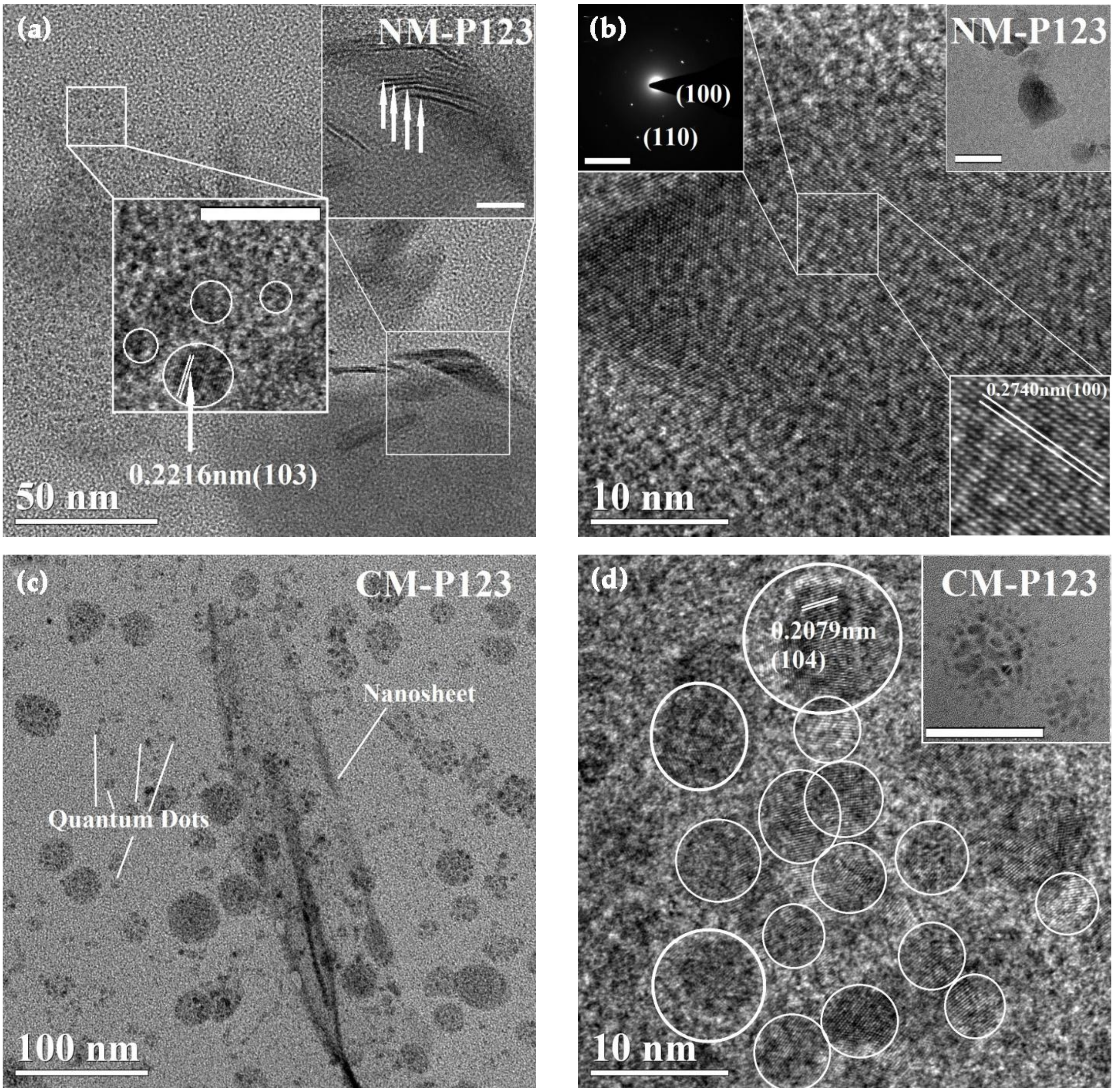

Figure. S4: TEM images of products exfoliated in P123 aqueous solution from natural molybdenite (a, b), commercial MoS_2 (c, d). Inset in (a): magnifications of the selected area; insets in (b): a SAED pattern, scale bar=5nm-1, a low resolution image, scale bar=100nm; inset in (d): a low resolution image, scale bar=20nm.

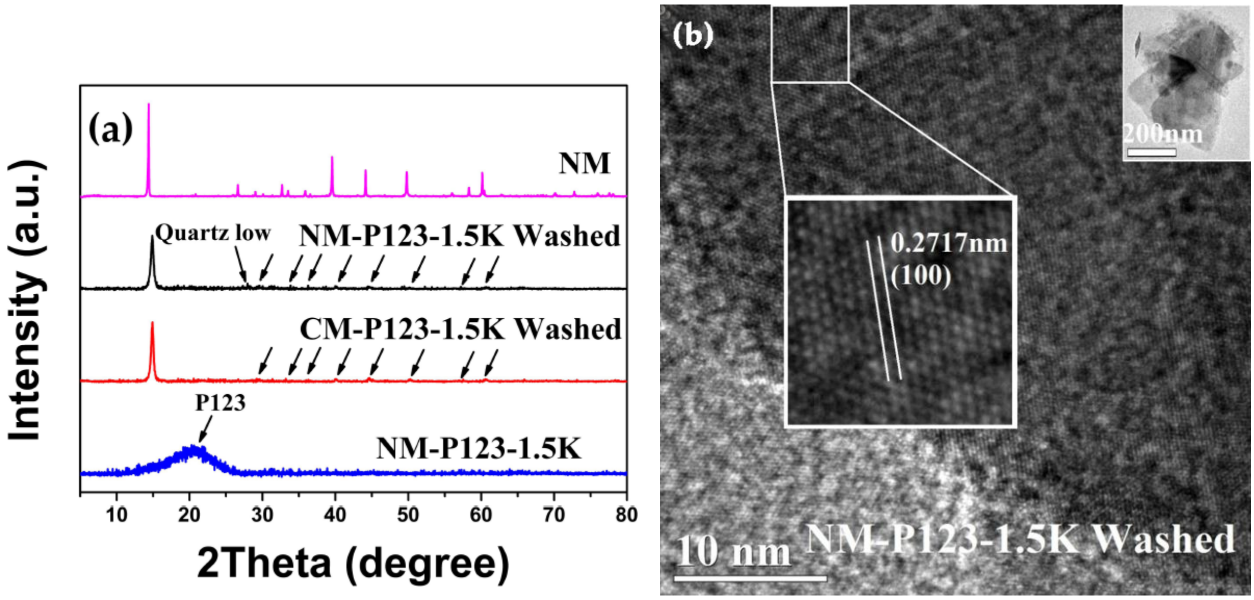

Figure. S5: (a): XRD patterns of products exfoliated in P123 from natural molybdenite and commercial MoS_2. (b): TEM images of NM-P123 powder-sample obtained by washing with deioned water several times to remove P123 after centrifuging at 1500rpm for 45min. It is shown that1500rpm is not sufficient to completely separate the bulk material， and nanosheets are lost during washing.

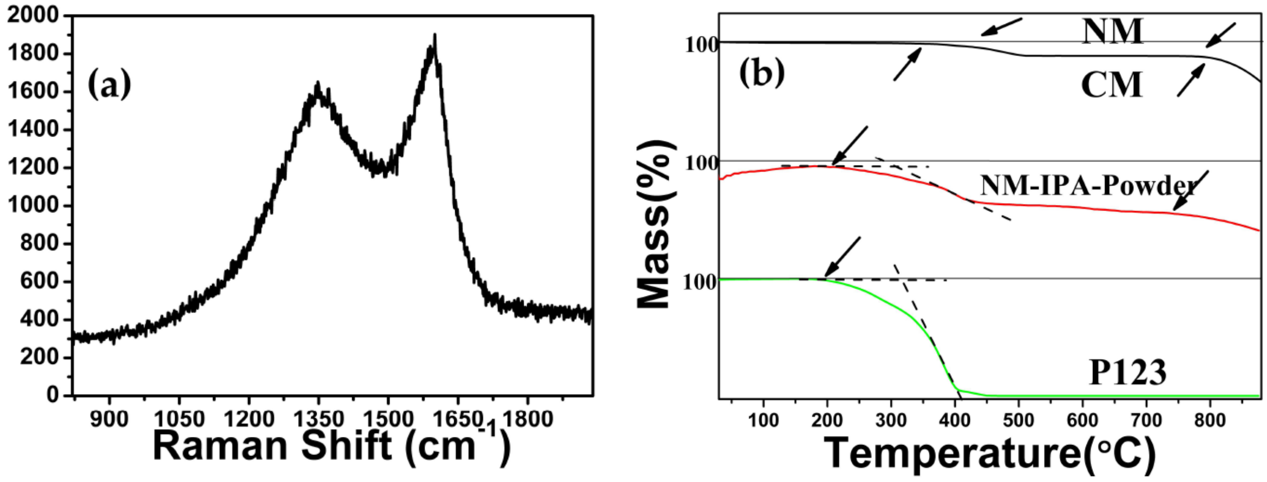

Figure. S6: (a)：Raman spectrum of NM-P123 powder-sample obtained by calcining at 450°C for 2h (no characteristic shifts of MoS_2 and many shifts of organic carbon were found). (b): TGA curves of NM, CM, NM-IPA-Powder and P123 (burning at 450°C could cause the change of MoS_2 nanosheets and introduce new organic carbon impurities).

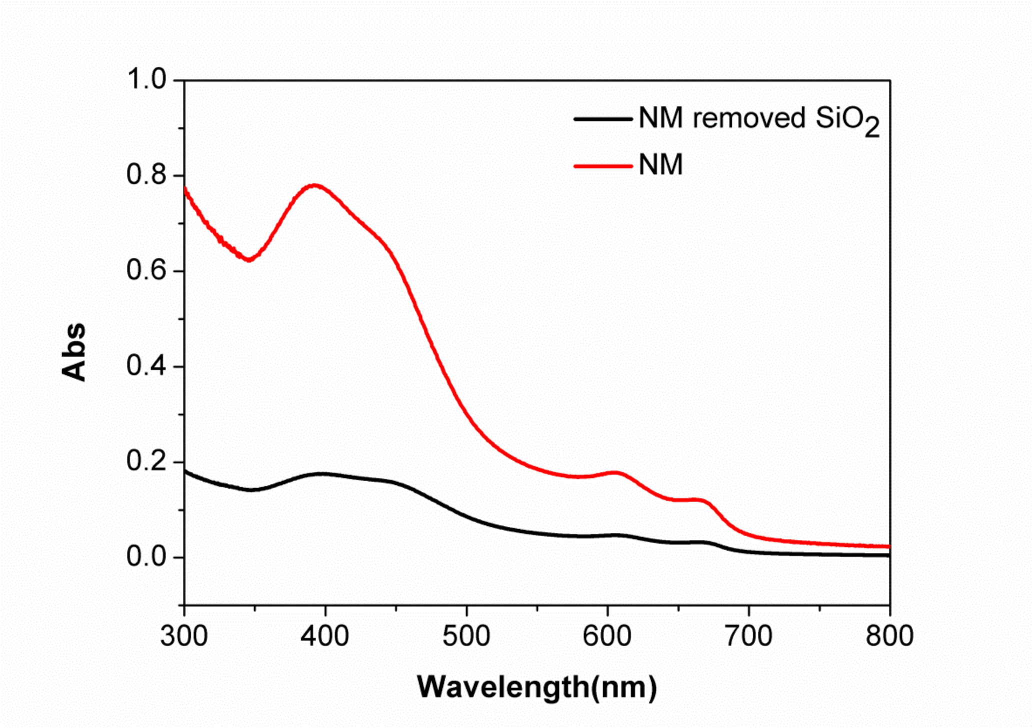

Figure. S7: UV-Vis absorption spectra stack plot of MoS_2 dispersions obtained from the initial NM and NM removed SiO_2.
